# Supplementary material for: Antennal Transcriptome Analysis and Identification of Olfactory Genes in Glenea cantor Fabricius (Cerambycidae: Lamiinae)
Source: Insects. 2022 Jun 17;13(6):553. doi: 10.3390/insects13060553 (PMC9224838; doi:10.3390/insects13060553)
Supplement: Supplementary file 1 [file insects-13-00553-s001.zip › insects-1763077-supplementary.pdf]

Figures

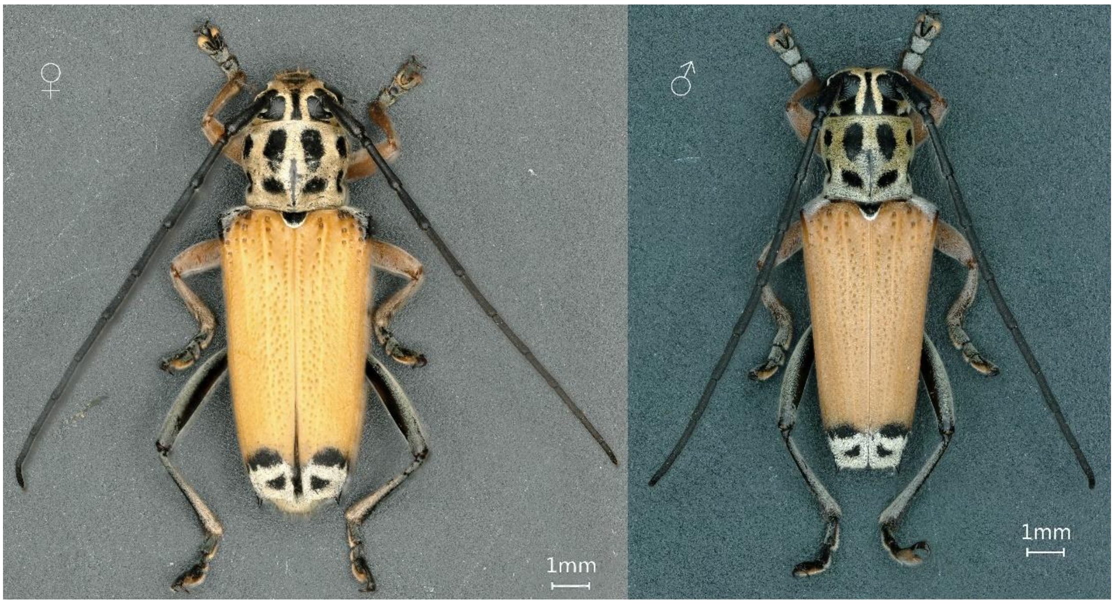

Figure S1. Adult male and female of *Glenea cantor*

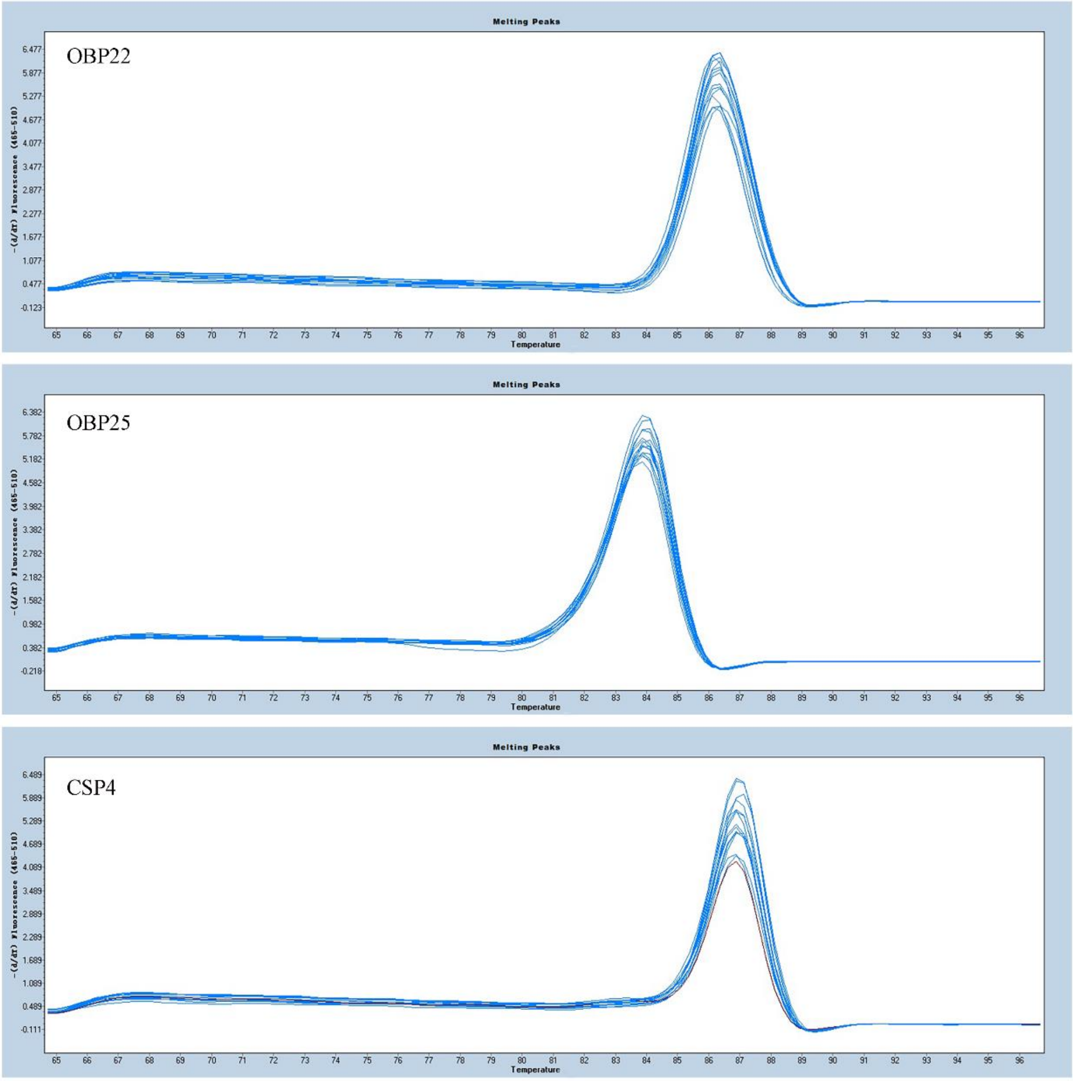

Figure S2. Melting curve of OBP22, OBP25, CSP4

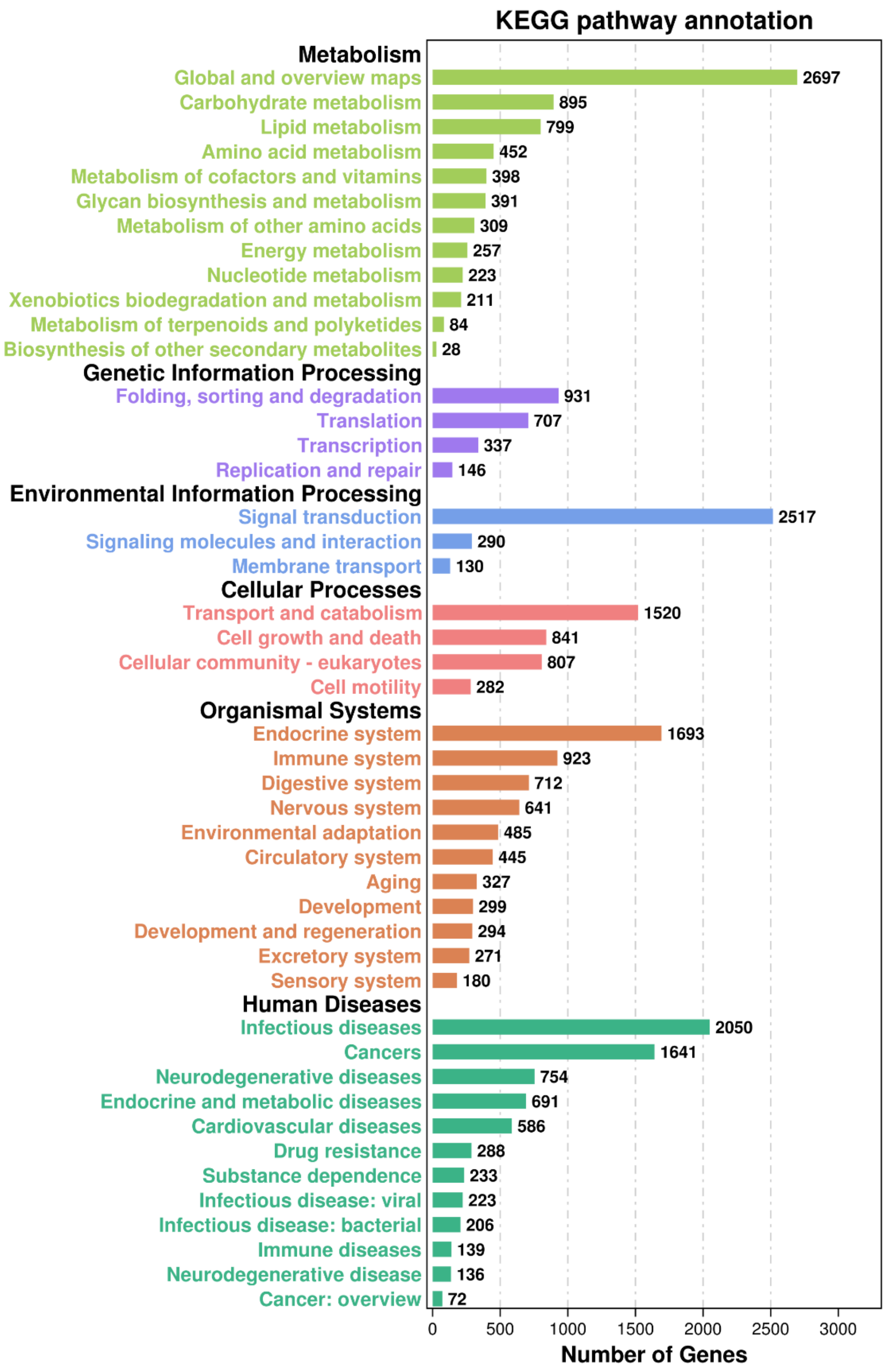

Figure S3. KEGG classification of *Glenea cantor* antennal transcriptome

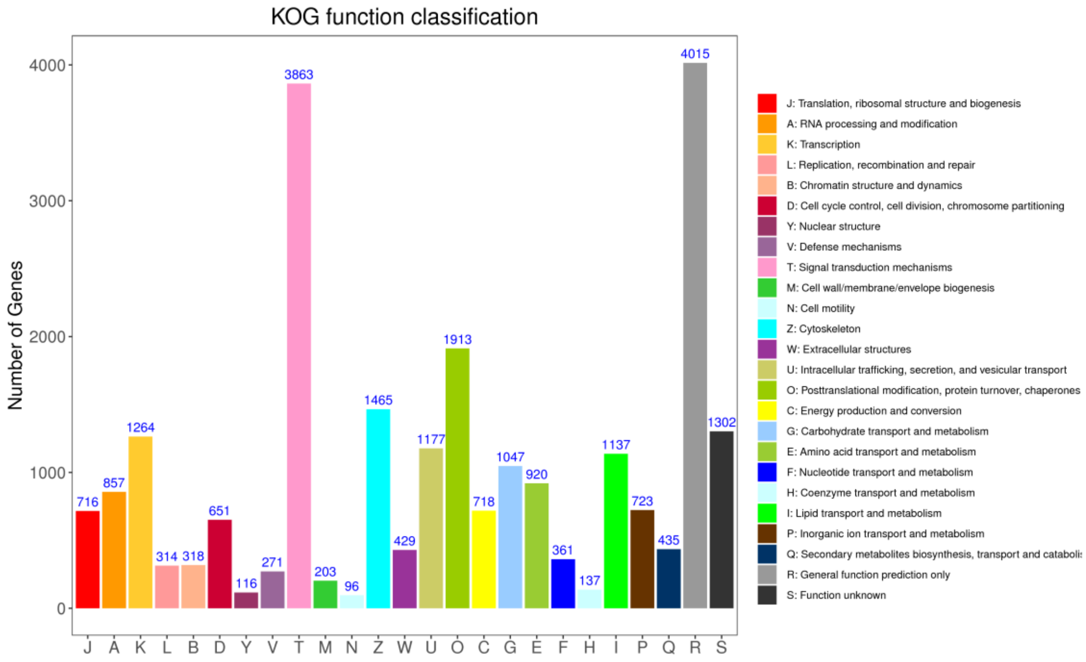

Figure S4. KOG annotation of *Glenea cantor* antennal transcriptome

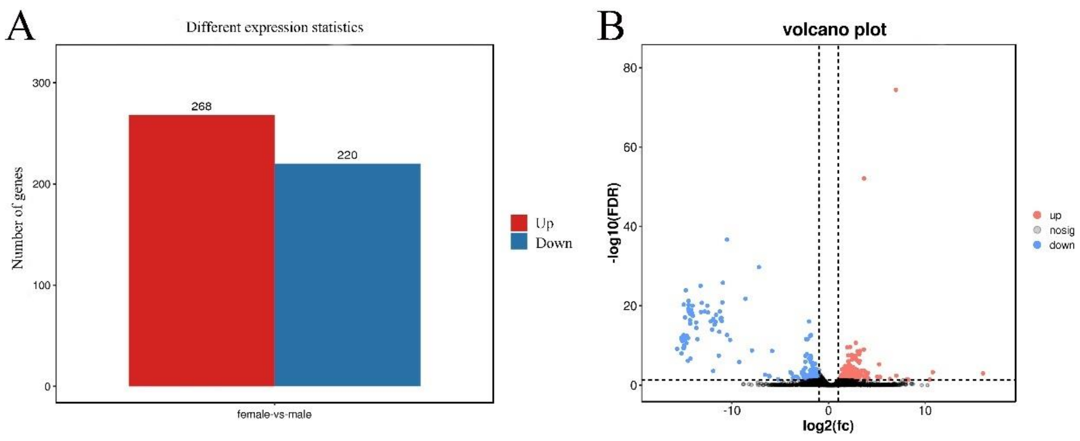

Figure S5. Differentially expressed genes in *Glenea cantor* antennal transcriptome. (A) Number of different expression genes statistics between sexes. (B) Volcano plot of female antennae vs male antennae.

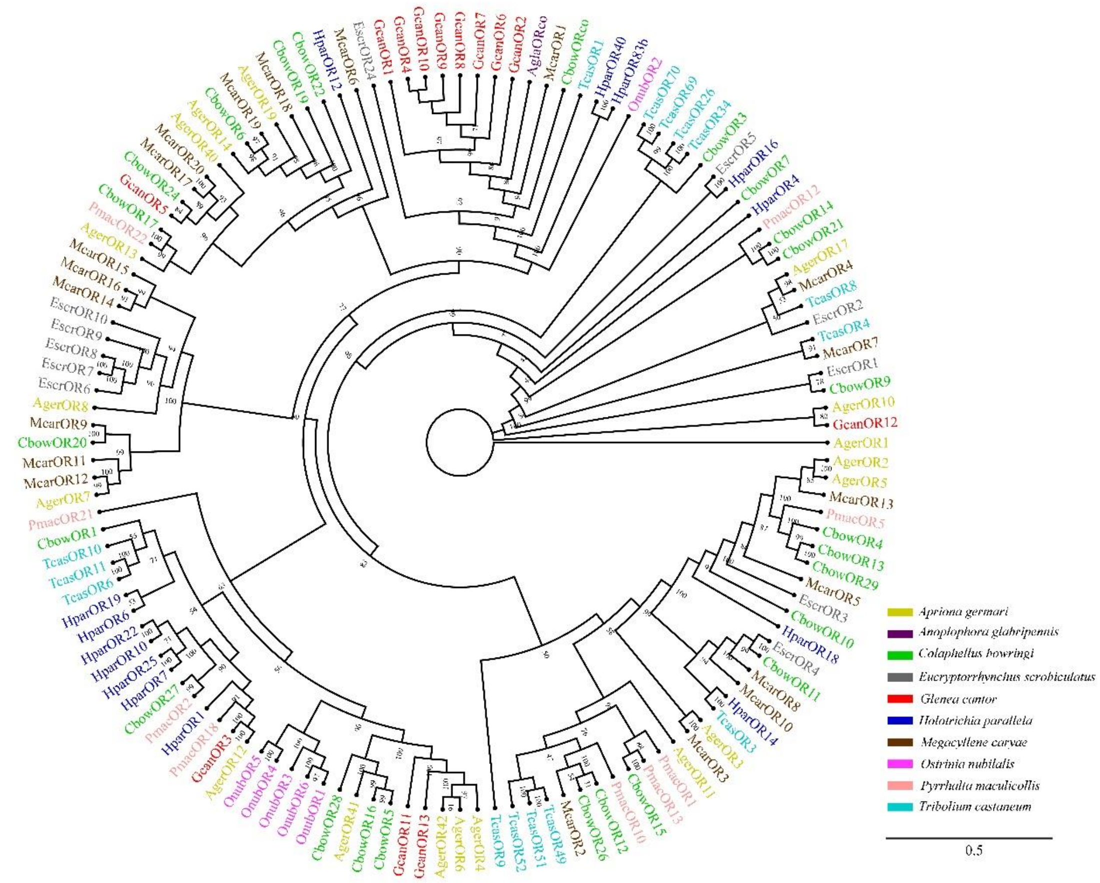

Figure S6. Phylogenetic analysis of insect odorant receptors (ORs).

Information of ORs was listed in Table S6. *Apriona germari* (Ager), *Anoplophora glabripennis* (Agla), *Colaphellus bowringi* (Cbow), *Eucryptorrhynchus scrobiculatus* (Escr), *Holotrichia parallela* (Hpar), *Megacyllene caryae* (Mcar), *Ostrinia nubilalis* (Onub), *Pyrrhalta maculicollis* (Pmac), and *Tribolium castaneum* (Tcas).

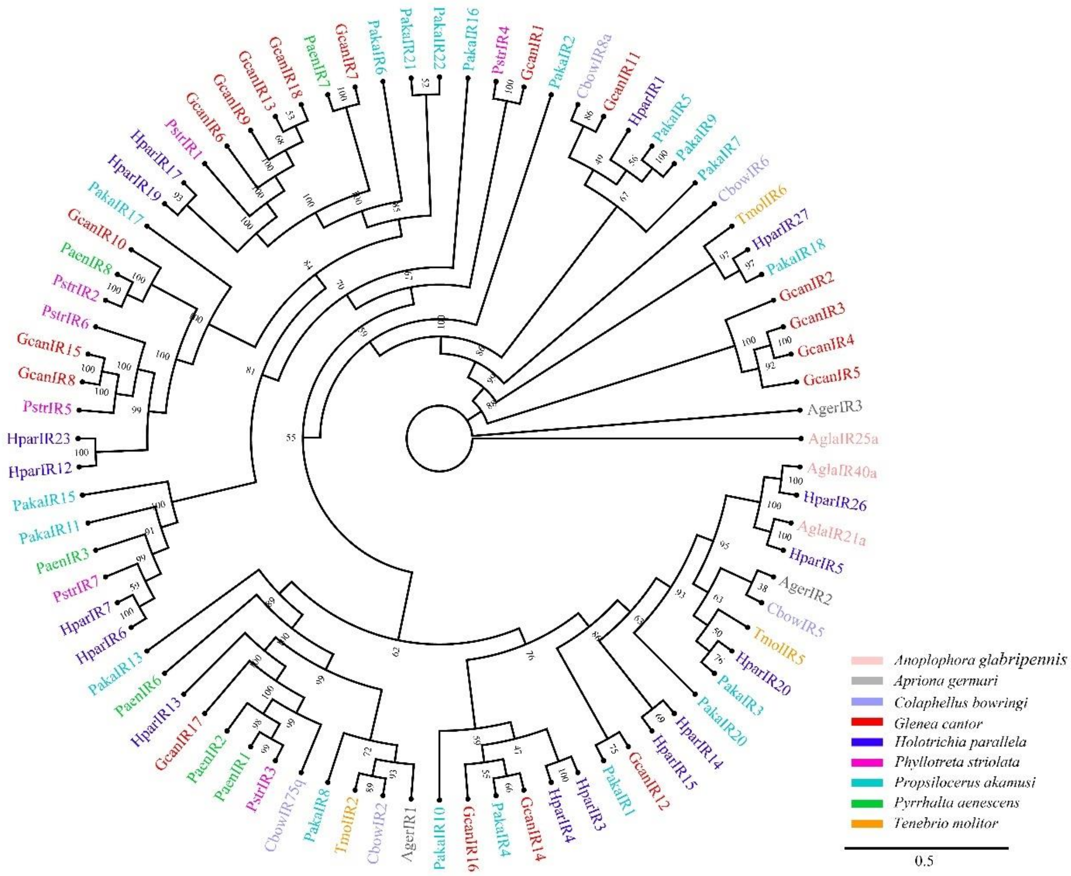

Figure S7. Phylogenetic analysis of insect ionotropic receptors (IRs).

Information of IRs was listed in Table S7. *Anoplophora glabripennis* (Agla), *Apriona germari* (Ager), *Colaphellus bowringi* (Cbow), *Holotrichia parallela* (Hpar), *Phyllotreta striolata* (Pstr), *Propsilocerus akamusi* (Paka), *Pyrrhalta aenescens* (Paen), and *Tenebrio molitor* (Tmol).

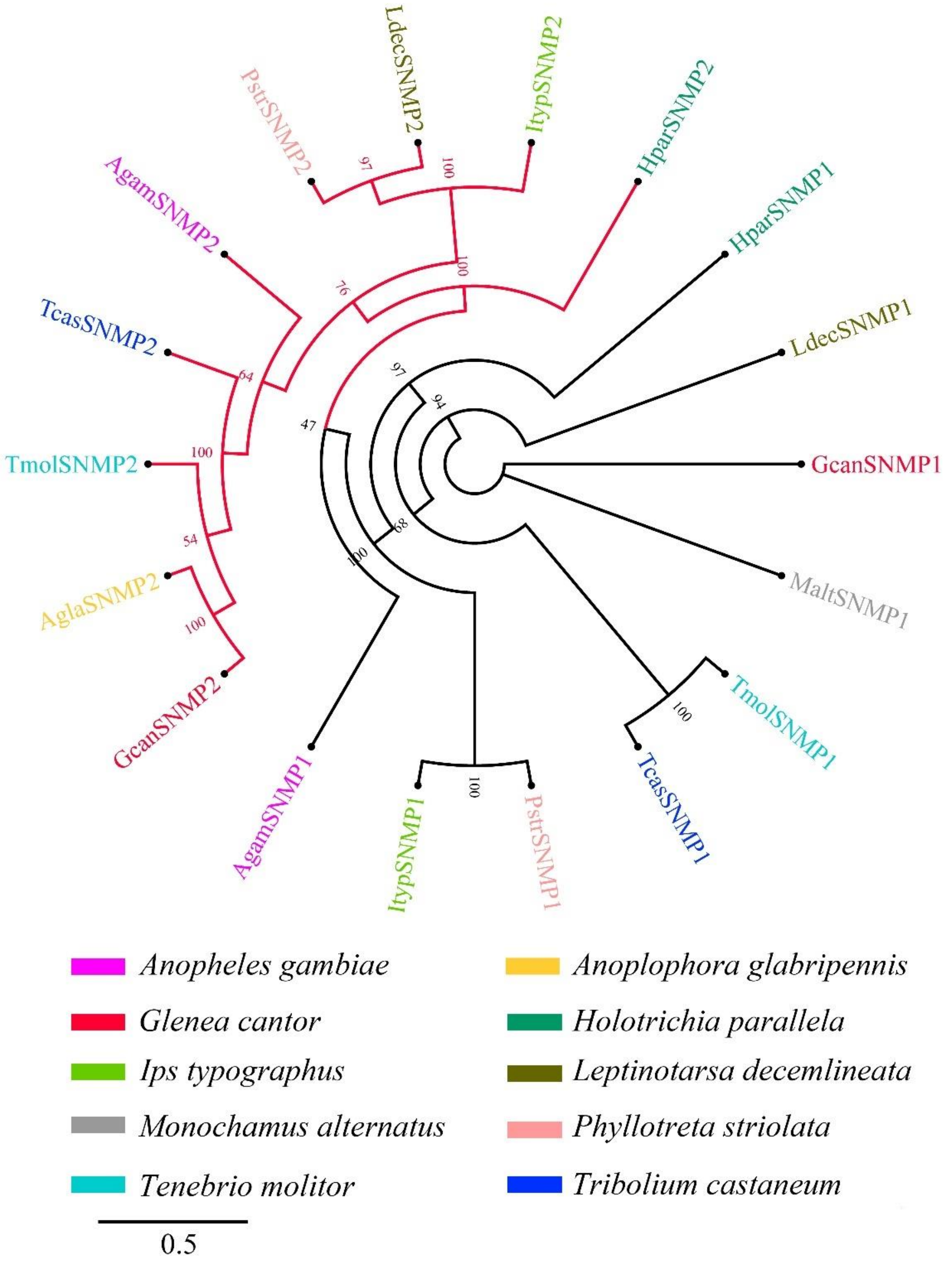

Figure S8. Phylogenetic analysis of insect sensory neuron membrane proteins (SNMPs).

Information of SNMPs was listed in Table S8. *Anopheles gambiae (*Agam), *Anoplophora glabripennis* (Agla), *Holotrichia parallela* (Hpar), *Ips typographus* (Ityp), *Leptinotarsa decemlineata* (Ldec), *Monochamus alternatus* (Malt), *Phyllotreta striolata* (Pstr), *Tenebrio molitor* (Tmol), and *Tribolium castaneum* (Tcas).

Tables

Table S1. Primers used in this study

Gene name	Primer sequences	Product length (bp)
OBP6	F: CGGATTCGTGACAAAGCTAAGG<br>R: AGGAAGCACTTTGCACCGTT	101
OBP8	F: GCTGAAGGTGGTGGATGGAT<br>R: CTCTGTTTCCTTCTCGGCGG	133
OBP21	F: GTCTGTTTGGCGTTGATGGC<br>R: GTGAACTCGCCCTTCCTAGC	143
OBP22	F: TTCACGGCGCATTCTTTGAC<br>R: GCTCTGCGAATCCAAAGCTG	135
OBP25	F: TTCACGGCGCATTCTTTGAC<br>R: GCTCTGCGAATCCAAAGCTG	135
CSP4	F: TGAGGTAGCGCTTGTCCTTG<br>R: GGTGGCCTTAATGGTCGTGA	116
CSP10	F: CTTTGAAGACCAACTGCGCC<br>R: GCCTCGAGTTCATTCCACCA	109
IR6	F: GGCCTCCGGGAAGAAATTGA<br>R: TCATGGGCTTGGAATCACCC	137
IR8	F: AAAAGCAGCAAGATTGGCCG<br>R: TCGTCACGGGATCGCTTATG	134
IR9	F: TTCTTCTCGTCCCATGCACC<br>R: AAGGCAAGACTCTGTACGGC	139
IR10	F: CCTCCGCAAACATCCTGACT<br>R: AAGCCGGTTAGGAAAGGTGG	125
IR18	F: ACGTCAAGTGTTCCTCCACG<br>R: GATTTGGCCAATGTCGGTGG	124

Table S2. Amplification efficiency and regression coefficient of qRT-PCR primers used in qRT-PCR

Gene name	Amplification efficiency (E)	Regression coefficient (R^2)
OBP22	94.39%	0.998
OBP25	100.19%	0.997
CSP4	91.99%	0.996

Table S3. Protein names and gene accession used in phylogenetic tree of OBPs

Name	ID	Name	ID	Name	ID	Name	ID
RferOBP1768	AVR54526.1	AcorOBP1	AKC58522.1	BhorOBP1	AHA33382.1	PmacOBP17	APC94208.1
RferOBP11	ANE37555.1	AcorOBP3	AKC58524.1	BhorOBP2	AHA33380.1	PmacOBP18	APC94209.1
RferOBP10	ANE37554.1	AcorOBP4	AKC58525.1	BhorOBP3	AHA33381.1	PmacOBP20	APC94211.1
RferOBP9	ANE37553.1	AcorOBP5	AKC58526.1	DponOBP1	AKK25129.1	PmacOBP22	APC94177.1
RferOBP8	ANE37552.1	AcorOBP6	AKC58527.1	DponOBP2	AKK25130.1	PmacOBP23	APC94180.1
RferOBP7	ANE37551.1	AcorOBP7	AKC58528.1	DponOBP3	AKK25131.1	PmacOBP24	APC94183.1
RferOBP6	ANE37550.1	AcorOBP8	AKC58529.1	DponOBP4	AKK25132.1	PmacOBP25	APC94185.1
RferOBP5	ANE37549.1	AcorOBP9	AKC58530.1	DponOBP5	AKK25133.1	PmacOBP27	APC94197.1
RferOBP4	ANE37548.1	AcorOBP10	AKC58531.1	DponOBP6	AKK25134.1	PmacOBP28	APC94187.1
RferOBP3	ANE37547.1	AcorOBP11	AKC58532.1	DponOBP10	AKK25136.1	PmacOBP32	APC94178.1
RferOBP2	ANE37546.1	AcorOBP12	AKC58533.1	DponOBP12	AKK25137.1	PmacOBP34	APC94181.1
RferOBP1	ANE37545.1	AcorOBP13	AKC58534.1	DponOBP13	AKK25138.1	PmacOBP35	APC94182.1
RferOBP3213	AVR54530.1	AcorOBP14	AKC58520.1	DponOBP15	AKK25139.1	TcasOBP1	EFA05678.1
RferOBP107	AVR54529.1	AcorOBP15	AKC58521.1	DponOBP16	AKK25140.1	TcasOBP2	EFA05676.2
RferOBP23	AVR54528.1	XquaOBP1	AXO78379.1	DponOBP17	AKK25141.1	TcasOBP3	EFA05675.1
MaltOBP1	ABR53888.1	XquaOBP2	AXO78380.1	DponOBP18	AKK25142.1	TcasOBP4	EFA05742.1
MaltOBP2	AHA39267.1	XquaOBP3	AXO78381.1	DponOBP19	AKK25143.1	TcasOBP5	EFA05677.1
MaltOBP3	AHA39268.1	XquaOBP4	AXO78382.1	DponOBP20	AKK25144.1	TcasOBP6	EFA04594.1
MaltOBP4	AHA39269.1	XquaOBP5	AXO78383.1	DponOBP21	AKK25145.1	TcasOBP7	EFA04593.1
MaltOBP5	AHA39270.1	XquaOBP6	AXO78384.1	DarmOBP1	AIY61044.1	TcasOBP8	EFA04687.2
MaltOBP6	AJO67868.1	XquaOBP7	AXO78385.1	DarmOBP2	AIY61045.1	TcasOBP9	EFA10713.1
AglaOBP1	ATG83411.1	XquaOBP8	AXO78386.1	DarmOBP3	ALM64965.1	TcasOBP10	EFA07542.1
AglaOBP2	ARU83753.1	XquaOBP9	AXO78387.1	DarmOBP4	ALM64966.1	TcasOBP11	EFA05695.1
AglaOBP3	ARU83754.1	XquaOBP10	AXO78388.1	DarmOBP5	ALM64967.1	TcasOBP12	EFA02857.1
AglaOBP4	ARH65459.1	XquaOBP11	AXO78389.1	DarmOBP6	ALM64968.1	TcasOBP13	EFA02858.1

AglaOBP5	ARH65460.1	XquaOBP12	AXO78390.1	DarmOBP7	ALM64969.1	TcasOBP14	EFA02914.1
AglaOBP6	ARH65461.1	XquaOBP13	AXO78391.1	DarmOBP8	ALM64970.1	TcasOBP15	EFA12066.1
AglaOBP7	ARH65462.1	XquaOBP14	AXO78392.1	DarmOBP13	ALM64971.1	TcasOBP17	EFA02861.1
AglaOBP8	ARH65463.1	XquaOBP15	AXO78393.1	DarmOBP14	ALM64972.1	TcasOBP18	EFA02860.1
AglaOBP9	ARH65464.1	XquaOBP16	AXO78394.1	DarmOBP15	ALM64972.1	TcasOBP19	EFA02960.1
AglaOBP10	ARH65465.1	XquaOBP17	AXO78395.1	PmacOBP1	APC94199.1	TcasOBP20	EFA05793.2
AglaOBP11	ARH65466.1	XquaOBP18	AXO78396.1	PmacOBP2	APC94200.1	TcasOBP21	EFA09215.2
AglaOBP12	ARH65467.1	XquaOBP19	AXO78397.1	PmacOBP3	APC94201.1	TcasOBP22	EFA09155.2
AglaOBP13	ARH65468.1	XquaOBP20	AXO78398.1	PmacOBP4	APC94202.1	TcasOBP23	EFA10803.1
AglaOBP14	ARH65469.1	XquaOBP21	AXO78399.1	PmacOBP5	APC94193.1	TcasOBP24	EFA04576.1
AglaOBP15	ARH65470.1	XquaOBP23	AXO78401.1	PmacOBP12	APC94192.1	TcasOBP25	EFA04747.2
AglaOBP16	ARH65471.1	XquaOBP24	AXO78402.1	PmacOBP14	APC94205.1	TcasOBP26	EFA04746.2

Table S4. Protein names and gene accession used in phylogenetic tree of CSPs

Name	ID	Name	ID	Name	ID	Name	ID
TcasCSP1	ABH88175.1	MaltCSP8	AIX97040.1	TmolCSP11	AJO62217.1	DadjCSP9	QPZ89241.1
TcasCSP2	ABH88176.1	DponCSP1	AKK25146.1	TmolCSP12	AJO62218.1	HcicCSP3	AWT23272.1
TcasCSP4	ABH88177.1	DponCSP2	AKK25146.1	DarmCSP1	AXF54070.1	HcicCSP6	AWT23265.1
TcasCSP5	ABH88178.1	DponCSP3	AGI05160.1	DarmCSP2	AXF53965.1	HcicCSP5	AWT23246.1
TcasCSP6	ABH88179.1	DponCSP4	AKK25148.1	DarmCSP3	AXF54071.1	HcicCSP4	AWT23245.1
TcasCSP7	ABH88180.1	DponCSP6	AKK25149.1	DarmCSP4	AXF54072.1	HparCSP17	AVM18966.1
TcasCSP8	ABH88181.1	DponCSP8	AGI05164.1	DarmCSP5	AXF54073.1	HparCSP1	AKI84384.1
TcasCSP9	ABH88182.1	DponCSP11	AGI05163.1	DarmCSP6	AXF54077.1	HparCSP2	AKI84385.1
TcasCSP10	ABH88183.1	RdomCSP1	AIX97109.1	DarmCSP7	AXF54074.1	HparCSP3	AKI84386.1
TcasCSP11	ABH88184.1	RdomCSP2	AIX97110.1	DarmCSP8	AXF54075.1	HparCSP4	AKI84387.1
TcasCSP12	ABH88185.1	RdomCSP4	AIX97112.1	AmalCSP1	AXG21594.1	HparCSP6	AKI84389.1
TcasCSP13	ABH88186.1	RdomCSP6	AIX97114.1	AmalCSP2	AXG21595.1	HparCSP7	AKI84390.1
TcasCSP15	ABH88188.1	RdomCSP7	AIX97115.1	AmalCSP3	AXG21596.1	HparCSP8	AKI84391.1
TcasCSP18	ABH88191.1	TmolCSP1	AJO62207.1	AmalCSP4	AXG21597.1	HparCSP9	AKI84392.1
TcasCSP19	ABH88192.1	TmolCSP2	AJO62208.1	AmalCSP5	AXG21598.1	HparCSP10	AKI84393.1
MaltCSP1	AIX97041.1	TmolCSP3	AJO62209.1	AmalCSP6	AXG21599.1	HparCSP11	AKI84394.1
MaltCSP2	AIX97042.1	TmolCSP4	AJO62210.1	DadjCSP1	QPZ89235.1	HparCSP12	AKI84395.1
MaltCSP3	AIX97043.1	TmolCSP5	AJO62211.1	DadjCSP2	QPZ89236.1	HparCSP13	AKI84396.1
MaltCSP4	AIX97044.1	TmolCSP6	AJO62212.1	DadjCSP3	QPZ89237.1	HparCSP14	AKI84397.1
MaltCSP5	AIX97045.1	TmolCSP7	AJO62213.1	DadjCSP4	QPZ89238.1	HparCSP15	AKI84398.1
MaltCSP6	AIX97046.1	TmolCSP8	AJO62214.1	DadjCSP6	QPZ89239.1	HparCSP16	AKI84399.1
MaltCSP7	AIX97047.1	TmolCSP9	AJO62215.1	DadjCSP8	QPZ89240.1		

Table S5. ORs, IRs and SNMPs identified in *Glenea cantor*

Gene Name	Unigene ID	Unigene Length(bp)	ORF (aa)	Complete ORF	Signal peptide	Transmembrane domain	Homology search with known protein				
							Name	Species	E-value	Accession	Identity (%)
OR1	Isoform0004381	1050	349	YES	0	4	odorant receptor 25	*Apriona germari*	0.00E+00	QNH68049.1	92.98
OR2	Isoform0007897	1119	372	YES	0	4	odorant receptor 25	*Apriona germari*	0.00E+00	QNH68049.1	97.15
OR3	Isoform0007991	1206	401	YES	0	6	odorant receptor 18	*Pyrrhalta maculicollis*	2.00E-106	APC94230.1	41.46
OR4	Isoform0008538	1440	479	YES	0	7	odorant receptor 25	*Apriona germari*	0.00E+00	QNH68049.1	94.36
OR5	Isoform0021309	987	328	YES	0	0	olfactory receptor 4, partial	*Monochamus alternatus*	8.00E-128	AIX97095.1	77.6
OR6	Isoform0009718	1440	479	YES	0	7	odorant receptor 25	*Apriona germari*	0.00E+00	QNH68049.1	94.36
OR7	Isoform0009738	1440	479	YES	0	7	odorant receptor 1	*Anoplophora chinensis*	0.00E+00	AVN97813.1	92.69
OR8	Isoform0012997	1440	479	YES	0	7	olfactory receptor 1	*Monochamus alternatus*	0.00E+00	AIX97092.1	93.6
OR9	Isoform0013465	1440	479	YES	0	7	odorant receptor	*Anoplophora chinensis*	0.00E+00	AUF73041.1	92.49
OR10	Isoform0013778	1440	479	YES	0	7	odorant receptor 25	*Apriona germari*	0.00E+00	QNH68049.1	94.36
OR11	Isoform0019959	1113	370	YES	0	6	olfactory receptor 9	*Monochamus alternatus*	9.00E-26	AIX97100.1	53.76
OR12	Isoform0021105	1179	392	YES	0	6	odorant receptor 4	*Anoplophora chinensis*	2.00E-45	AVN97816.1	43.24
OR13	Isoform0020476	1056	351	YES	0	4	odorant receptor 6	*Apriona germari*	8.00E-34	QNH68033.1	45.45

IR1	Isoform0005356	1734	577	YES	21	NO	ionotropic receptor 4	*Phyllotreta striolata*	0.00E+00	ANQ46496.1	83.51
IR2	Isoform0005426	2181	726	YES	26	2	ionotropic receptor 25a	*Anoplophora glabripennis*	0.00E+00	XP_018574744.1	92.09
IR3	Isoform0005476	2793	930	YES	26	3	ionotropic receptor 25a	*Anoplophora glabripennis*	0.00E+00	XP_018574744.1	92.77
IR4	Isoform0006107	2739	912	YES	0	3	ionotropic receptor 25a	*Anoplophora glabripennis*	0.00E+00	XP_018574744.1	92.95
IR5	Isoform0006436	2637	878	YES	0	3	ionotropic receptor 1	*Phyllotreta striolata*	0.00E+00	ANQ46493.1	59.4
IR6	Isoform0006446	2757	918	YES	23	3	ionotropic receptor	*Anoplophora chinensis*	5.00E-85	AUF73080.1	74.87
IR7	Isoform0006954	2808	935	YES	21	3	ionotropic receptor	*Anoplophora chinensis*	0.00E+00	AUF73077.1	72.8
IR8	Isoform0007217	2877	958	YES	20	3	ionotropic receptor	*Anoplophora chinensis*	0.00E+00	AUF73087.1	76.14
IR9	Isoform0008252	1455	484	YES	0	3	ionotropic receptor	*Anoplophora chinensis*	0.00E+00	AUF73078.1	78.51
IR10	Isoform0008721	2517	838	YES	27	2	ionotropic receptor	*Anoplophora chinensis*	0.00E+00	AUF73070.1	74.01
IR11	Isoform0010036	2661	886	YES	22	3	ionotropic receptor 25a	*Anoplophora glabripennis*	0.00E+00	XP_023311227.1	74.66
IR12	Isoform0010563	2568	855	YES	19	4	ionotropic receptor 93a isoform X1	*Anoplophora glabripennis*	0.00E+00	XP_018576792.1	83
IR13	Isoform0010576	2253	750	YES	0	3	ionotropic receptor	*Anoplophora chinensis*	0.00E+00	AUF73078.1	73.33
IR14	Isoform0011329	1899	632	YES	20	3	Ionotropic receptor 571	*Blattella germanica*	3.00E-37	PSN50089.1	25.42
IR15	Isoform0012510	2193	730	YES	0	3	ionotropic receptor	*Anoplophora chinensis*	0.00E+00	AUF73087.1	78.86
IR16	Isoform0012892	1827	608	YES	0	3	Ionotropic receptor 442	*Blattella germanica*	8.00E-06	PSN29401.1	20.93

IR17	Isoform0015821	1896	631	YES	19	4	Ionotropic receptor	*Anoplophora chinensis*	0.00E+00	AUF73085.1	69.2
IR18	Isoform0020397	924	307	YES	33	2	ionotropic receptor 1	*Phyllotreta striolata*	1.00E-129	ANQ46493.1	66.56
SNMP1	Isoform0017326	1356	451	YES	0	1	sensory neuron membrane protein 1	*Monochamus alternatus*	0.00E+00	AIX97076.1	80.89
SNMP2	Isoform0019652	1548	515	YES	30	2	sensory neuron membrane protein 2	*Anoplophora glabripennis*	0.00E+00	XP_018566911.1	69.19

Table S6. Protein names and gene accession used in phylogenetic tree of ORs

Name	ID	Name	ID	Name	ID	Name	ID
AgerOR1	QNH68028.1	EscrOR9	QXE93190.1	OnubOR1	ADB89178.1	CbowOR1	ALR72546.1
AgerOR2	QNH68029.1	EscrOR10	QXE93191.1	OnubOR2	ADB89179.1	CbowOR3	ALR72548.1
AgerOR3	QNH68030.1	PmacOR18	APC94230.1	OnubOR3	ADB89180.1	CbowOR4	ALR72549.1
AgerOR4	QNH68031.1	PmacOR1	APC94224.1	OnubOR4	ADB89181.1	CbowOR5	ALR72550.1
AgerOR5	QNH68032.1	PmacOR2	APC94225.1	OnubOR5	ADB89182.1	CbowOR6	ALR72551.1
AgerOR6	QNH68033.1	PmacOR5	APC94229.1	OnubOR6	ADB89183.1	CbowOR7	ALR72552.1
AgerOR7	QNH68034.1	PmacOR22	APC94232.1	TcasOR70	EEZ99312.1	CbowOR9	ALR72554.1
AgerOR8	QNH68035.1	PmacOR10	APC94237.1	TcasOR69	EEZ99311.1	CbowOR10	ALR72555.1
AgerOR10	QNH68036.1	PmacOR12	APC94239.1	TcasOR49	EEZ99303.1	CbowOR11	ALR72556.1
AgerOR11	QNH68037.1	PmacOR13	APC94240.1	TcasOR51	EEZ99302.1	CbowOR12	ALR72557.1
AgerOR12	QNH68038.1	PmacOR21	APC94243.1	TcasOR52	EEZ99301.1	CbowOR13	ALR72558.1
AgerOR13	QNH68039.1	HparOR40	AVH87281.1	TcasOR26	EEZ99239.1	CbowOR14	ALR72559.1
AgerOR14	QNH68040.1	HparOR1	AVH87242.1	TcasOR34	EEZ99230.1	CbowOR15	ALR72560.1
AgerOR17	QNH68042.1	HparOR4	AVH87245.1	HparOR83b	AEG88961.1	CbowOR16	ALR72561.1
AgerOR19	QNH68044.1	HparOR6	AVH87247.1	TcasOR4	CAM84002.1	CbowOR17	ALR72562.1
EscrOR24	QXE93205.1	HparOR7	AVH87248.1	TcasOR3	CAM84001.1	CbowOR19	ALR72564.1
EscrOR1	QXE93182.1	HparOR10	AVH87251.1	TcasOR6	CAM84004.1	CbowOR20	ALR72565.1
EscrOR2	QXE93183.1	HparOR12	AVH87253.1	TcasOR8	CAM84006.1	CbowOR21	ALR72566.1
EscrOR3	QXE93184.1	HparOR14	AVH87255.1	TcasOR9	CAM84007.1	CbowOR22	ALR72567.1
EscrOR4	QXE93185.1	HparOR16	AVH87257.1	TcasOR10	CAM84008.1	CbowOR24	ALR72568.1
EscrOR5	QXE93186.1	HparOR18	AVH87259.1	TcasOR11	CAM84009.1	CbowOR26	ALR72569.1
EscrOR6	QXE93187.1	HparOR19	AVH87260.1	TcasOR1	EFA05687.1	CbowOR27	ALR72570.1
EscrOR7	QXE93188.1	HparOR22	AVH87263.1	AglaORco	XP_018568191.1	CbowOR28	ALR72571.1
EscrOR8	QXE93189.1	HparOR25	AVH87266.1	CbowORco	ALR72547.1	CbowOR29	ALR72572.1

Note: several sequences used here are not available in NCBI and they were listed below.

>McarOR1
ATGCTGAAATTTAAGGTGGTCGGCCTTGTGGCCGACCTCATGCCCAATATAAGGCTAAT
CCAGGCGTCTGGACATTTTATGTTCAACTACCATGCAGACAATTCGGGGGCTTTACACA
CACTGAGGCTGGGATACTCTTGCATGAATCTTGTCTTCGTATTGTTACAATATGGGGCCA
TTTTCGGGAATTTAGTCGCAGAGAAGGATGACGTCAACGATTTAGCAGCCAATACCATC
ACGGTGTTATTTTTCACACATTGCGTTACCAAATTCGTTTACTTTGCCGTCAGATCAAAA
CTATTCTATAGGACCCTAGGTATATGGAATCAGGCCAATAGTCATCCGCTTTTCGTGGAG
TCTAACAACAGGTACCATGCTCTTGCATTGAAAAAGATGAGAATCCTACTGATATGTGT
TGGGATGACAACAATTTTGTCAGCTGCAGCTTGGACTGGAATAACTTTTGTTGGAGAA
AGTGTTCATACAATAAAAGACCCCAACAACGAAAATGAAACTATTACTGAGGAAATAC
CTAGACTCTTGATAAAATCGTGGTACCCATGGGACGCCATGTCAGGAATGGCATACTAC
GCCAGTCTGGTGTTTCAAGTCTACTATGTTTTCTTCTCCCTTAGTCAATCCAATCTCCTG
GACAGCTTGTTCTGTTCTTGGCTCATCTTCGCTTGTGAGCAGCTTCAGCATCTAAAGGA
GATCATGAAACCTCTAATGGAACTATCTGCTTCATTAGACACCTACGTACCAAAAAGCG
CTGACCTCTTTAGGGCTCCAAGTGCCACCTCTCAAGATAATCTCATAGAAAATGAATAT
AACGCAAAGAACGAAGAACTGAATCTAAAGGGTATCTACAATACGAGGCAAGAACTT
GGTGGCCATTTTCGTTCAGGAACTTTGCAAACGTTTGGTCAAGGGGGTGGAGGTGTTG
GTCCAAATGGTTTAACGAAGAAACAAGAGCTTATGGTGCGGTCTGCTATTAAGTACTG
GGTAGAGAGGCATAAGCACGTTGTTAGGCTTGTTACTGCTATTGGAGATGCATATGGTG
TAGCCCTTTTACTCCATATGTTGACTGCCACCGTTATGTTGACGCTGTTGGCATACCAAG
CTACCAAGATAAATGGAGTTAATACGTATGCTGCTTCTGTGATAGGCTATCTGGTCTATT
CTCTGGCACAGGTGTTTCACTTTTGCATATTTGGAAATAGATTAATAGAAGAGAGTTCA
TCTGTGATGGAGGCAGCTTATAGTTGTCACTGGTATGATGGTTCAGAGGAAGCCAAAA
CTTTTGTACAAATCGTCTGTCAGCAATGTCAGAAAGCAATGTCCATATCTGGTGCTAAG
TTCTTTACAATTTCACTGGATCTTTTTGCATCAGTACTTGGTGCTGTCGTGACTTACTTC
ATGGTGTTGGTCCAGCTCAAATAA

>McarOR2
ATGAAAAGAAACATGAGTTACGACAATTTTGATTACACAGTTTTCTTCACTCACAACAT
TTTGATGTACAAAATCTTTGGGTTCTGGCGACCTGATGATGACATGAAACGTGAAAAA
CTATACAACTGTTACACTCTGATATGCACGATTATATGGCTATTATTTTTGGCATCCCAAT
ATATCTTCATAATCACTAACATCCAAAATGTTGACGAAGTAACAGCCACATCGTTCGTA
ACAATTACATTTTCTATAAATCTGATTAAAATGTTAGCCATATACAGGAATATGAACAGG
ATAAAACAGTTGATAAAGGATATGAACCTACCAATGTTTCAAGCAAAGTGTGCAAGAC
ACAGAGACATTATTGATTACACCAGAATTTATACTATTTTTTTCTATATCTGTCTCTATTTT
GGGAACACAGACAGACATTATTTTTGGACAATTGTTCCCTTTATAGGTGATGAAAGGGC
TACTTTAACTCATGGATGGTTTCCTTATAATGAAACTAAATCGGTTAACTACGAAATAAC
CTACGTATTCCAAACGACTGTGAGTGTTTGGAACACGATGCTTTGTCTAAATCTGGATA
CTTTTACTGGTTCCTTACTAATTCTAATTGGTCTACAATGTGATTTGCTGTGTGTTACATT
GGAAAACTTAGGTGATTTCCATGTAGAGAATGGAGTATTGTGCGAGAATTCTGAGGAAT
ATCAATCAAGTTTGGTTAATGATAAAGTGAAATTTTCGAAAACCATGACGGAAAATTTG
GTTGTTTGTATCAAGCATCACAAAGAAATTATGAGAGTATCAAAAGACGTAGAAGATAT
TCACAGGGTAAGCGTATTCATCCTCTTTCTAGGAGGTGCCTTGATAATGTGTTGCTGTTT
GTTTCAGTTATCTGTGGTCCCCATTGGTTCTATTGAGTTCTTTATGTTACTATTTTTTCTA

ATCTCTATATTAACTGAACAATTTATATACTGCTGGTTTGGAAATGAAGTAATACAAAAG
AGTTCCAGGATACTACATTCTGCTTATTGTACACCATGGCTTGATTGCGATATAAATTTCC
AGAAAGTTCTCCTACAACTGATGACGCAAACATATCGGCCTATCACACTTAAAGCTGGC
GGACTCTTCACAATATCTATATCAGTATACATATCAGTAATAAGAACCTCATATTCTTATTT
CACTTTACTGAAAAAATAG

>McarOR3
ATGTCACAAAAGGTGGACCCACAGTATTTTAAGAAGCACTTGAAATGGCTAACTTGGC
TTGGAATCGACATCATACCCATAGAAAAAGTGTGGTACGCGATACCATACAAATTGTAT
GCTTTCGTCCTTTTGGTATATGTGTACTTATACAGTCTATTAGAAATTATTGATATTGTCA
AGTCTTCGGATTTTAACAGCATGACGTTTGGTTTGAGCTACTCGGTTACTCATATCTTAG
GTGCTGCAAAAATTACCATACTGATACTTAAGAAAAAGATTCTTCGTGATATGTTAATCA
GATTAGAACAGGGTTACTTCGTACCTAATAAGGCAAGAGGTGGTGAAAAGGAACAAC
AGTTAGTTAATGCATCTGTTATAAGAGCAAATTTACATGCAGATATATTTAACACATTGG
TCTATCTTATAATTGGTATTCGCTGTCTCTACGCAATTTTTGATAAAGGAGTTTACGTGGA
GGTTCTAGATGAAAAGCTTAACGTGACCACGTTGAAACATATAAGAACTCTCCCCTATA
AAGCTTGGTTGCCTGTAGACTTGAACAAAAGTCCCGCTTACGAGTTCATGTTCATCATA
CAAGCATCTTGCTTGGTACTATACGGATATTATATTGGTTTTCTAGACTCTCTGATATACG
GGATGATGATTCACATGAATAATCAGTACTTAATACTCAGGAACATCCTAGAGCACTAC
GTAGAACTCGCAAAAAAATATTGTCCTGAACAGAAACCCAAATTCAGTTACAGATGATA
CATCAACGGACTATATTAAACTACACAATGGAATAGAAAGGCAGAAGACATTGGCTGG
ACCTGTTTTGGATGTAATAGAAAATATAGCATATCATTGTGCAAAATACCACCTAGCCAT
TATTGACTATTGTGATGATATAGAGAAAGAATTTAGTAATTTGATGCTTCTCCAGTTTCT
GTCCAGTCTTTACATTTTATGTTTTCAATTATTCCAGCTTAGTTTGGTTACGAATTACTTC
AGCTTCGATTGTATAAGCATGTGCCTCTACTTGATCCTTATGATGTACCAACTCTTTTGTT
ACTGTTGGTACGGAAATGCAGTTATGATCCAGAGCCTGGACATTTCCTCAGTCATCTAT
AACACAGATTGGTTGGTAACGAATGAGAGCACAAAGAAATGTTTGTTGCTGATGATGA
TGCGAGCTCAAAGGCCTATTATCTTTACAGCAGGAAAGTTTGCTTTCCTCTCCCTCCCA
ACGTATATGGCGATTGTTCGAGGTTCTGCATCTTATTTTATGGTACTACAACAAATGCAG
TAG

>McarOR4
ATGGCTCCATCTTATGTGTTTGATCTTCCCAAAGCTTTTGAATTTGAAAAGAAACTCCTT
CTATATACAGGATTATACCCCAACACAGGATTGGTTAACAAGTATATTTATTACTTGTCAG
GACTTTTTCACATTGGAATAACGATTCTAATAGAAATTTCACTGATTATAGTTATATCAAT
CCACATAGATAATTTGTCAACTATAACAGATGCCTTAATGTTCTTTGTAACGCAAATTGC
TTTGACTTGGAAGCTTACCAACGTGTGTATAAAGAGAAAAGTATTTTGCGAAATAGAA
GAAATACTCAGTCAGCCAATTTTCTACAATTTATCACAAGAATGCGAAAACATAATTCA
TTACTATGTCAAATTCTCTCACCGTTTTGCCAGATGTTTTAGAATTATATGCATTATGGTA
TGTGCCACTAATGGTACTTTGCCACTGGTGGGAGGTAAGCTAGGCCACGCTATGCTACT
AGGTTGGAACCCATGGGATAGTGAAGATCGGATAAAATATTACCTAAACTCCACTTTTC
AACTAACAGCCTTATGCGTAAGTGCTTGCATTAATTCCACCATTGATATCCTCACTGTTA
TATTACTAGCAATTGCTACTGCTCAAATAGAAATTTTGAAGAATAATTTGGTTAACATTA
AATATGGTGAAAAAGAAGCTAAGAAACTGTTTAATGAAAATGTTCGCTTACATTATGAA

ATATTAAGGTTTGTAAATGCCGTAGATCGTAGCCTTTCCTCAGGAATTTTAAGTCAAATA
TTTGGGAGTGTATTAGTAATATGTGTCACATGTTTCCAGCTTATCATTGTTTCAGTACAA
AGTATACAGGGGGCTTTCCTCCTCATTTACCTATTGTGTATGACGTTCCAAGTGGGGCTG
TACTGTTGGTTTGGGCACTACCTTATAGATAGTAGCGACACCATAATTCAAGCTGTTTAC
ATGTCGGATTGGTACGAAGCAAACAATAGTCTTAGAAAAGCTGTCATAATATTCATGGA
AAGATGCAAGCAACCCATCGTCTTACGCATCGGCGGCTTATTTCCATTATCTCTAGGAA
CTTTTACTAGCATAATGCGGTCATCCTATTCATATTTCGCAGTACTAAGGAAGTGGTACG
AACCAGAATAA

>McarOR5
ATGACGGACAAAGGATATACTCCCCATTTTTTTCGAACAAATGAAATAATCGAAGTTTA
TACAGGAGCGTGGATGTATAACGAAAATTTAGTGGCTCCTGGAAAAAAGTGGCTGCTT
TATATATGGAGTGTCTTGATATATATAGGGGCGGTATTTTTTCTCTTCTTGGAGTTCTTGA
AACTACGTGATACGATGAAAGTTTCCAACGACTTCATTCGCCAATGTGGACTCATTTCC
TGTCACAGCTTGTGTGTAGTAAAATTTGTGATTTTAGTACTGCGACACAGAAAGATTAA
AAGACTTATGGATACATTGCAAGATAAAAAATACCAATATGAACCTTTAGGAGACTTTA
GTCCTGGGCAACGTTTTGATGAAGCAAGGAAACTTACCCATTGGTGCACAATAGGCGT
CTTCTGTTTATATTCTTGCGCAGCTGTATCTGCTCATATTTCAGCGGAAGTCTTAATTAAT
AAAGACGCAAAACGAGAACGATTTGATGGAAACATAACTTGTTACGAATACATGACCT
TTTATTTTGCTATTCCATTTCCTTCGGATACCAAAGCTCAGTGCGAAATGAGTTTTATTTT
CATGCACTTTTGCATCGACATATATGCTTGGTTCGCTGCAGGACATGACAGTTTTTATGC
AGCCTTACTTAACTGTCTGAGAGTTCAGGTGGACATACTGTGTGACGCTTTTAGGACA
ATCAGACCGAGAGTTTTGAAACGACTTGAACTACCTCAAGATTTAAGTATATTTCATGA
CGACGATTTTCCCAAACTGGAGGAAGCGCTTTATCGAGAACTAACGCATTTAACAGAA
CACTTGATGATTTTGCTTAGAGTTGCGGATGACCTTGAGGAGGTTTTTAATTTGATCAC
ACTGGCTCAGACAGTATCGTCTTTGATCGTTTTCGCATCCTGTTTGTTTATTACATCCAC
TATTCCACTTTCTTCTCCAGAATTTTTCGCTCAAGTGGAATATTTTACATGTATGCTAATA
GAGTTGAGTCTATTTTGTTGGTTTGGAAGTGCAGCAACCAGAGCGAGTGAAGCTATAT
CGCCGGCAATATATGAATCTGATTGGTATGGAACCAGCAAGAGATTCAAGCAATCTGTG
TTGATAATAATGTGTCGAATGCAAAACCCAATATATTTATCAATTGGAAAATTCTGTCCT
CTAAAACTTGACACCATTGTTATGGTCTTCAAGTGTTCCTTTTCGTACTACACCGTATTT
AAAGCAGTCGGTGAATAA

>McarOR6
ATGCCTTTGTCAGTTTTCTTCATTTATTTTTTAATAATTCTCAGTTCTATGCAGCCATTTG
CAGCCATAGCGTACCAATTTTACGTTGGGATTGAAGACATGAATATCATATCTGAAGCGT
TCATAGGCATCAGTGATTTGGTCGGTTTTCTCTTTATTTATATTTGCTTCCGCAAACATCG
CGGACTTATTAAGGAAACCATAAAAGCGTCGGCAGTATTTCTAAAATACTGTAGTCCAA
ACGTAATGGAGAAAGCAGAAGAGGAAGTTCAGACGTATACAAAAGGGCTTTTAATATA
CTTCAGCATCGGCCTTACATTCAACGGACTGATTCCTTTATATGACTACGAAAACTGTG
ACCAAAGACGTCTGTCCGACTACTACCGAGCGCACGATCCCTGTGGTATGCCCATCAG
GATTTGGGTCCCTTTCGATGCCAGGAAACCAGTCATATACTATTTGGTATTTTTTTTGCA
CGCCAACGCATGCTTAAACATTTGCTACGGAGTATTATGTATAACGATGACACTAGTCG
GTCTTTTAATACACATAACAGCTCAAATAAAAAACCTGAGGCAGAACCTTTTACAAGTG

TTCGATGAGTTACCAGAAGATGGTGACTGTTATTCCGAAACGTTAGTTTTAAAGTTAGA
AAATAAACTGAAGTTCTGTGTTAAATATCACATCATAATTATCAACTACACAGATCAAGT
ATTCGCCGCTTTCAACCTAATGCTCATAGTCCACATATCCCTTACGTCCCTGATATTCGG
CGTCTTGGGCTACCAGATAGTCACGGTGGAAGACTTCACCGAGAAACTGCGTTACGTC
ATGCACCTGGGCGGCTGGATTGCCCTGTTGTTCCTGACGTGCTATTACGGCCAATTGAT
TTTAGATGAGAGCACCACCGTAGCCAACGCCGCGTACCAGAGTAAATGGTACAACGGC
CCGACGTACCTAAGGAAGAATTTATGTTTGATCATCATGCGATCGCAGAAACCCCTGAA
GCTGAGGGCGGCTTCCATAGGGGTGATTTCTCTGGAGACGTTTCTTTCGGTGATAAAA
ACAGCGTATTCATACTTCGCCTTACTTCTCAGCATAGCGGAATAG

>McarOR7
GTGTTGCTATACTGTACTACAGAATTCGCCTTTCTTTGTAAATTGATGAACTTTGTTTTA
AGTAAAAAGGAGATCATTGAATTGGAAGCAATATTGGAAAGCCGATTGTTTACCGTAG
ATACACCGGAGGAAGAAGCCATTATAAAAACATCAACAAGGCAAATAAGAAAGCTAGC
CAATATCTATAAAACATTATGTTTCCTATCTGTGACATTCTATGCTCTATTTCCGCTGGCA
GATGGCGGTCGAGAAGCTCAAAAGCTTCCGTTACCGGGGTGGTTTCCGTTTAATGTTT
GTAACCACTATTATGAAGTATTTATCTTCGAAGCAATTGGAATAGGCTTGTGTGCCTGGT
TTAATTCGGCACTAGACCTGTTAGTTGTTATAATGATGATTTTGGGAAAGGCTCAGTTTG
AGCTTTTGCGTCATAGGTTGATGAACATAGCAATTTACGGTGAGGATGGAGAAAGACG
AAGAAGAGTCAAAATGTGCGCCCAACATTATAAATCTATACTACGCTTTGTGATGTTAA
CAGAAAGCATCTACTCGAATGGTATTTTTGTTCAGTTCATGAGCAGCGGTATAGTAATTT
GTTTTACTGGATTCCAAATGCTTATCATCTCATTAAAAAGTATTCAGTTTGTCCAGAGGA
TACTATATCTTAGTTGCATGATGTATCAAATAGTGATGTACTGTTGGTATGGTCAAGTACT
TACGGATAGCAGCAACAAAATTACGGAAGCTTGTTATTTGGCCGATTGGATTAATTGTA
ACGTAATTTTGAGAAAATCGTTGCTCATTATAATGGAGAGAGCCAAATATCCTGCGAAA
ATTAGAGCTGCAAATATTTTTACAGTGAACTTGGAAACGCTATTAACTATTTTAAGATCC
TCCTATTCATATTTTGCATTGATATATAGCATTTATGATACTAAGAATGAAACTAAGTGA

>McarOR8
ATGACGGTACCATATGCAGACGACTTTTTCCATACCAACCGTTGGATACTCTATATTGGT
GGCCTTTGGTGGCCTGACACATACAAAAGCATTTACCACAAGATCCTCTACATGTCCTA
TTGCGCTGCTAATTTCCTATTTTGCAACCTGTACTTTACACCCACCGAAGTCTTAAGCTT
GGCTAGCACCTACAAGAGCATATACCACTTGATCAAGAACTTCAGCCTCTCGCAGATGC
ACGTACTAGGGTTCACCAAGGTCCTTTTTTTCGTATTCAAGGGGTACAAGATGAAAGC
GATAATAAGCGTTTTGGAAGACAAGAAGTTGCACTACGAAGATTGCGATGAAGTGAAT
TTCCATCCTGGTATGCTAACTAATAAGTACAAGAAGATTGGAAGGGTGGCTGGTATTAT
ATATCTAGTGCTGCCGTTGGTAGTAATCCTATTAGCGTACACGCTAAGTGCTATCGCTGC
TCTTAGATATGTTGAAGGTGACTCAAATCATCAGCTACCTGAAAGGTTGCCTTTCTACA
GTTGGATGCCCTTCAGTTATGATACTCCCAAAAAGCATTTGATTGCACTGGTGTATCAA
GCTACTCCGCTGGTTTCATATTCATTCAGTGTTATTGGTATGGATTTTTTATTCGCCAATA
TAATGAACTGCATCGCTATGAATTTTACCATCATACAACAAGCATTCCGAACAATTAGAG
AACGTGCTGCTATTAGGGTTAAAGAACCGTTGAAAGTCAAAGACGAATTATACAATTCT
GAACCATTGCAAAGGGAATTAAACAAAGAAATGAGAAAAATTATTCAGCACTTACAAA
CTGTGTACAGGATGTGTGATGAACTAGAGGACGTGCATAAGTATCTTACGTTGGCACAA

ACGTTATCGCAACTGTTCATCCTTTGTGCCTCTTTTACCTAACCTCAATTACCCCCTTT
GGTAACCAATTGGTGATCGAGGGCATATTTATGATTATGGTTATATCTCCAATAGTGTTTT
ATTGTTGGTTTGGAGACGAAGTTACTCATCAGGGTGGTGAAATATCTGTTGCTATATGG
CAAAGCGACTGGTTGGGTGCAACGAAGTCTTTTAAAACCTGTATGATTATAAATATGAT
TAGAACTCAGAAACCTGTCTATTTGACTACAGGAAAGTTTGCCCCACTCACACTTGCTA
CTTTAGTATCCATATTCAAAGCATCTTACTCTTTCTTCACAGTTTTAAAAAATACCAGCA
ATCAATGA

>McarOR9
ATGACTATATTGGGTATATGGCCAGTAAAAGCTAACTGGTTCAAAAAATTGAGACTCTA
CGAAGTTTACCATCGAATTTCTTTTTGGATACTTTTTGAGTTTTATTATAACGCTGATAAT
AAAAATTTTGTACAGGTAATGGGGAAGAACTTCTCCGAAACTGCAGAAGTTCTTGGTG
TAGTAATCGTTCTCCTTATCACCTCCTTCAAGGTCAAAATATGCACCTCACCCAAAATCA
AAAACTTACTCCAACAAATTGAAGACGCCGAAAAGATTATTATAGAAACAACCGAATT
GGACACCCGCAATATTTACAACCAACATATTAAGATGTCCACCAAAGAGAACACTGTC
CAGTTAATGATCGGAGTTCTAGCAATATCCCTTTACTCTGTTCGACCTATCTTGGCAAAC
CGGGGTTTAGAACCGGAAAGCAAAAACAAAATGTTTATATTCGCTTCTTGGTTCCCCTT
CGACGAACAGACCTACTACGCTCCTGCCTACTTGATTCAATTCATATCGGGGCTGTACA
GCACTGGTTACACTATATCCACCACGATGTTTCTGTTCAACGCTATGATATTCGCAAGAT
GCGAAATAAAAATTCTACAAAACCAGTTCGTAAATTTCACTTATTACGTGAAAAAGGAT
GCCAAGGATAATTGCAGGACTTACGAGGAGAGTCAGAAGGTTGCCCTAGAAGACCGC
ATTTTGAAACACAGGAAAATTATAAATTTTGTTAACACGTTGGACACTTCTTTCAAGAC
CATTTTGTTGTTGGACTTCACAGTAACATCCTTCCAGTTCTCGATGGTGGTGATCCAAAT
GGTTCAGCGGTCTCAGCTGGATGTCGCTGTTGTATCGATGGTTATGTATCTTAGCACATT
AGCCCTGCAACTGTATTTAGTATATAGCAACGCTCATGAAATTATCATTGAGAGCAATAA
AATAGCGCAGGCTGTATTCGAAAGTGAGTGGTACGATCTACCAAATGATGTAAAGAAG
GCTTTTGTTATTATTATGCTAAGAGCACAAAAACCGTTGTACTTGTCTATAGGTCCCTTA
TATCAGGTCAGGTGTGATATGTTATTCAAAATTTTGCATGCATTGTATTCATATATTTGTC
ATTTTCTTGAAGTAAATTATATAGTAACTATTGTTTAG

>McarOR10
ATGAGCAGTTACCCAAAAAAATTATTTTTCCTAAATCGTTGGATTTTGTGTTGCGTTGGG
ATGTGGCCCCCCGACAACCAAAATAAATTATTTCGCTTTCTCTATAAGGTCTATGCTATC
GCCGCCTTTTTTTATATAATGGTCCTCTATAACGTTTTGGAGATTATCAGCCTCATTTATA
CGTACAATGACACAGTTTCATTCATGAAAAATGTCAGTGCGGTGTGTGTCCATTTAGCA
GGAGCAGCCAAGTCGGTTATTTTTTATTTGCGTGGTGATAAGGTAGTTGAAATGATGAT
AACTTTGGAGAGTGAAGAGTTAAGATACGAAGATTGCGAGGCAAGAAATTTTTATCCA
GGAAAAATTTCCAAATCCTGCAAAATTACTGTGGCGAAATTAACGGCGTTGTGTTTCGT
CATGGTCCACATAGTGCTGCTGTCATCTTTTATACCCCCTATTCTACAGATATTATTATGT
ATTATCAAAAGGGATGCAACAGTACTCCCAGATCGTTTGCCATACCTTATCTGGATTCCG
TTTAAGATGGATACCGTTAGTCGTTTCACTTTGGCCCTGGTGTTCCAAATCTTTGGCATG
TTTGGTGGAGCATACAATATATCTGGAATGGATTCAATATTCCTGGGCCTAATGCACTGC
ATATCGCAGAATCTGGTCATAATCCAGGGCGCCTTCCTGACAATCAAGGAGAGGTCCGT
GAAAAGGATTAAAGGTCCGGCCCTTGCCGCTGACAGACTGAATAATTCCGAGTGTTTG

AATGCCGCCATGAATTCGGAGATGAGGAAGGTCTCCCGGAATCTGCAAACAATTTTA
ACGTATGCTGGGATCTAGAAAGAGAATATAAATATTTGATGTTGTTACAAGTACTTATCA
CGTTACTCATCCTCTGTTCCAGTCTATATACATTTTCATCGGCCACTCCTAATAGTAAACT
GTTTTATACAGAAATAATTTATATATCAGCGATGATGTTTGAACCATTTATGTACTGTTGG
TTTGGAAATGAAGTTACACATAAGGCTGACGAAATGAGTAATTCTGTATATCAATGCGA
TTGGTTAGGAACTGACAAAAAATTTAAAACCAGTCTAATTTTAAATCTGACTAGATCGA
TGAAACCTATCTACTTGACTGCGGGAAATTTCGTACCTCTTACACTTGCTACATTCGTAG
CGGTTGTAAAAGGTTCCTATTCCCTTTTCACAGTCATCAAGGGTAGCAACTAA

>McarOR11
ATGTACTTCAACTCCCTCCTAGGAGTCTGGCCCTTCGTATTCGAAAGGCCAGACTTCCG
TCTGTGGCAGATGATGTACAAGATGTACTCTAACCTCATGTTGGTCTTTGGCACATACG
TTATTTGTACCCAATATACACAGCTAGTTATGCTGCTGCAAGAAGAAGAGATATGGGTA
CAAGAAATCATACGGAATCTATGTCTTACTCTGCTACATTCTATGGGATTGGCTAAGGTA
TATGCTATCAGGTCTGATAATTTGAAGGAGCTCATATCGGAAGCGTTGAAGGTAGAGGA
AGATATATACAGGCGTGGAGACGAGGATATTATGGAAATATACAGGTTATATGCTTGGCA
TAGCAGAGTTTCTAATATCGCTTTTCTCATCAACATTGCAATAGAGACGTGCTTCTACGC
TATGCACCCCCTCTACGTGGGAGAATTACCCCATTTCGACAAGGCCACCAACCAGACG
AAGATGATAAGGGCCTTGCCCATGTCTGCTTGGGTGCCCTTCGACATCCAGGAACAGT
ACCTTGAGGCATACCTTTGGCAGAGCGTCGAAGGGACAGTCACCGCTTCTTTCGTGAT
GTACACGGACATCTTCTCATTCAGCCTGATCATATTCCCTCTGGGACAGATCAGTATACT
GAGTCACGTGCTCAGAAATTTCGACCACTACGTCAAAAAGGCCCAGGAGAAGCACGG
GTGCGACAGAGATGAGGCTAGTTTCTTTATTGCCAGGGAATGCGTGGTGAAACATCAG
GATATAATTCGGTACATATGTGTTTTTAATAACGCAATGAAATACATAATGGTGTTCGACT
TCTTACAAAGTTCGATGCAATTGGCCACGATTGTAATACAACTTTTTGGGTCTGAACTC
AAAATGGTAGAGGTGATATTCCATGGTGAATTCGCCTTCTGTATGCTGATGAGACTTATG
GTATATTACTGGTACGCCAACGAAATTATGTTGAAGAGCTCAGACATAACCCTGGCTATT
TGGGAAGGCGTGTGGTACGAAGAGTCCCAAAGGGTGAAGCATATGATGTTGATGATTA
TAAGGAGGAGCAACAAACCGCTAGCTTTGGATATAGGGCCCTTCAGTACGATGACCCT
ACAGGCACTTTTAGGGATATTGAAGGCCACCTATTCATACATGACGATTATGTACAACC
GTTGA

>McarOR12
ATGAGGGCCAATGCCCTTCTCGGAGTTTGGCCTTTCATCTTTGAAGACAATCCCAAGTT
ACAGAAGATTTATGACGTATATTCAAGATGCACATTTATTTATTACCTACTCTTCATAATA
ACCGCCATAATTAAACTAATCTTTCTCATCTGCGACGAAGTTTTCGTGATCCAAGAAGT
TATTGCAAATTTATGTATAACCCTTCTGTATTCTGTGACTATTATGAGAGTATGGGCCATT
AAAACACCTAGAGTGAAGAATATAATTCGGGAAATAATTATTACGGAGGAAAGGATATT
GAAGTCCAAGGATGAAACAGTAATTACGATATATAATTCACACGCAATGCAGAGTAAAG
TTTCCAATATAATTTTCCTGGTCAATATTTTCTTAGTTACTGCATTATATTTCATTCATCCT
TTATATGTGGAGGATCGTGCGAAGTTTTACGAAAGCAAAAATATCACCGTGATAGAGAA
ACCATTGCCACTTTCATCCTGGTTTCCATTTAACGAGCAAGAACACTATTTGGTAACATA
CCTGTGGCATGTCTTGGACGGTTCCATAGGAGCATCTTTTGTTACCTACACCGACATATT
TACATTCAGCCTTATAATATTTCCGTTAGGCCAATTGAAAATATTGATACACATCATGTCT

AATTTTGAAAAATACGTAGATAAAATCCAGAATCAACTGGATTGTAGTCCAGAAGAAG
CTAGCTTTACCACGTTGAGGGAGTGTGTTTTAAAACACAATGAAATTATTAAATACATA
AACGACTTTAATACTGCTATGAGGAACATCATGGTTCTAGATTTCTTGCAGAGCTCAATA
CAACTAGCCTCAATTGTGTTACAACTTCTCGTGGCAGAATTTACTATATTAAATTTTGCA
TATAGTGGACAATTTGCCCTTAGTATGTTTATAAGGCTCCTGGTTTACTACTGGTATGCA
AATGAAATAATGGTACATAGTTCAGACGTCGCATTTGCTTTATGTACTAGTAACTGGTAC
GAGCAACCAGAAAAAGTGAAGAAGATGCTTGTAGTAATTCTGATGAGGTGTAATAAAT
TTCTCTGTTTAGAAATTGGACCATTTACTACTATGACGTTGGGTACATTTTTGGGAATAC
TCAAGGCAACGTACTCCTACATGATGGTAATATACAAATAA

>McarOR13
AATACGGTTACTAGTTTTAAGGAGGACTTCTTTCATGCTAACAGAGTGATATATAGAATA
TGTTCATTATGGCTACCAGGGAAAGAGATACCTCTACAACTAAGGGTCATGTATTTGAC
CTACGTGTTCGCCTGGTACTTCTTGTTTCTGACTTTCCTTATATGTGAGTTCCTAATCTTC
AAGGATATGCTACAGGAGGTCAGCAAATTCGTCAACTACTTTGGCATGTTATTCACCCA
CCTGGTTGGTACGCTCAAATTATCGGTTATAATACTGCAGTACAAGCGGATCAACAACC
TAATGAGTATTCTGCAGGACCCGGAGTATTGTTATGAGTCCTTGGGCGACTTTCAGCCT
GACGTACTACTTCACAAGAGCAAGATAATTAGTTTTATCGTATCTGTTTCGACTTTCGTT
TTATACAGTTTTGTTGGCATTTCCGCTCACATCAGCTCGCATATCGTCATGAACCAAGTA
GTGAAGAATCCCACTCTGGAGAAAAACATGAGCTGTGTGGACTTCGTACCATACTATTT
CTATACTCCCTTTACTGGAACAACTAAGTTGCAGTGTGAATCCATGTTTCTGCTTATGGA
CATATGTTATTTCATCCATGCCACAATAATTGCCTGTCATGATGGTGTCTTCGCGGGCCTT
CTCAACTGCCTAAGAACGAAACTGGTCATATTAGGTGGAGCATTCAAAACTATTAGACC
AAGATGTCTGAAAAGGCTTAATATGCCTACCAACTTTACTGTACTGCATGAGGAAGAGA
ATCCAGAGATCGAGAAAGTATTATACGCAGAACTGAATCATTGCATCAAGAATTTACAT
ATTCTTTTGCAGTCCCGTGATGATATTGAACATTGCTTCAGCTATGTGACCCTCGCACAA
TCTTTGGCATCTTTGTTTATACTGGCATCCTGTCTATACAACTCATCGACGGTGCCGGTA
ACTTCTCCAGACTTTTTTTCTCAATTAGAATACTTTGTATGCATTTTAACTCAACTCTCG
CTTATATGCTGGTTCGGTAACGAAATAACTTTAGCTAGCAATCATATAATACTGTCTTTAT
ATGAAGGAGATTGGTTTAGCGCCAGCCCAAGATTCAAGCGATCTATGATATTGACCATG
TGTCGTATGCAACGACCCCTGTATCTGTCAATTGGAAAGTTTTCACCACTTACTTTGGC
AACTTTAGTAGCGGTATGTCGAGGTTCCTTCTCATATTTTGCAGTTCTCCAGAGCATCTA
A

>McarOR14
ATGGATGGTGGTATATTGCGAGTTCAAAAACTGTTCATGATACTTTCAAACAAATGGGA
GGTTAATACAAAAAGTGTACTTATCAACAAACTCTGTAGAATACAGGGCATATTTTTTG
AATCGTACTTTATTCTTTTCACGCTTTATCTACCGTTCAATTTATTATTGCATAGGAAATG
TATGTTAATATTTTATGAACTCGGTGGCTATTTCTTGCATCACACGAATATAATTATCATG
AATATATTGTTCAGAAAAAATGCAATGAAGAAAACACTGAAGTACATTAAGAATTACGA
ACAAGTAGAATATAGCAAAGAGACTCAAGACTCGAAGGATATATATGGTTATTATTCGC
TGCTAAATGCTAGACTAGGCAAATATGTAATTGTGTTCGGTACTTGTATAGCAGGCATAA
GTTGGTATGTGTCAACAATTAGTTATTCCATCAAGGAGAATACAGAAGATTGCGCTGTT
TTGGAAGGCGTAATGTATCAAGTTTGGTATCCATTCAAAACACGGTATAATTGGTTGTCC

ATTATATTTGATTTATCTATGGCGTACATCGCAGTATCTATGCATATTTTTAATAGAATGTC
TCCTATAACGCTAGTGCTATTTCAACTGGCTCATATAAAGATTTTAGCAAATAAAATTAG
AAACATTGATACCCATGCAGAAGAATTGGCAAGTCTTCACGATGTCAACATTGAACAA
GCTTTAAATATAGCTGTTGACGAGTGTGTCAAAAGTCACCAAGAGGTTATGAGCTTAAT
GGATCTTCTTCTACAAGCAACAAAGGAAATGATGCTGATTGGATTCTTCAGCAGCTCTA
TGGAACTAGCGTCATTTATTATTCAATTATTCACGGCAGCCTCAAAGTACCATTTTATTC
GCTGCTTTATAATTTTTCCAATAGATCTTTTACAAATGTTAGCATTTTTCTGGTTTGCAGA
TGAAATCTATGTGGAGAGCACAACTTTGTCTAATGTTATTTACAATGAAGTAGATTGGA
CTCGTTATACCAAACCTTTGCGAATGAAATTGATTGTTATGATGATTGTTGCTCAAAAAC
CAATATATTTTAATGCTACTGGTATTGGAGAAATGACACTGGAGAAATTTAAATGTATCC
TCAATAGCTGTTTTTCTGCAGTGACATTTTTCCAAACGATGTATTATAACTAA

>McarOR15
ATGGAAAAACTAGTGCATATTAAATTACTAAGAAGGATGATGATAATTTGTGGTCAGTG
GAATTTCAAAAACTATAACAATCCTGCATTAACTATCTACAGAGCATACTCNCGTTTCAT
TATATACCACGTTATATTTATGACTCAAATGATATTATTGACTATTGCGATGCAATGGGAC
TGCAGGAGCAGAGTGATAGAAATGTTAATGCTACTATATTCAATACACCAACATTTAGT
GATGATTTTTCTGACAAAGATCTATAATCTGGAAAAATCTCTTAATTATATGATGGATTAT
GAAAGGGTAAAATTAAAACAGGCGGGTGAAGACGAGAAAAATGTTTACTTTAAGTATG
CCAGAGTCAACAATAATATGAACGTATTAATAATAATAGTTTGCATTTTAACTGCTATTAT
GTGGTATATGACTTCAATAAGGAACACTTTTACTGTTAGAGGAAATGAAGTATGTCCTAT
ATCAAAAGGACTCGTGTACCAAATCTGGTACCCGTTTAATTTCGACAACCAGTACTGGT
TAATAGTAATTAACGATTTAATATTTTTCCTGAATGTGGTCATCCTTCTTACATACACCAA
AATTATATCTATAACAGTGACGATATTCATGTTAGGCCAAATTAAGATTCTGCAGGAGAA
AATTCGAAATTTAGAACAAGATGCTTTGGTGCTACAAAGGATAAACCGAACTGAATATG
ATGAGTCACTGTTACTATCTCTAAAGCTTTGCATAAAAAGACATCAAGAAATTGGATGG
TTTATGGAAGTGCTGCAGGATTCTACAAGCTCAATAATATTGACACAATATTTTAGTAAT
ACTTTTGAAATGGCAGCTTTCTTAATACAGATGCTAACAGAAAAATCATTGTATCTTATA
ATACGATCTTTCATAGTATTTTGTATGGTTATACTGCAAGTCTACATATTTTATTGGTTCGC
AAATGAAGTCCAAATTGAGAGTACTGCAATTCCAGATATTATATATAGCGAAACTAAATG
GACAGAAAATGATCAAATAAGAAGGTATTTGCTTCTAATGATGACAAGATCTCAAAAA
AAGTTATCTTTTAAATCAGCAGCTATTGGTGACATGTCTCTGGCAACATTTACAAAGCTT
ATAAAACTATGCTATTCTATAGTAGCTTTCTTCAGAACAGCCTATGATTTGTGA

>McarOR16
ATGAGTGGCCAAACGCAAAATATATTTTTGCATGCGCAGAGGTACATTATGATATTTGTT
GGAAAATGGTACATAGACTTTGGAAGCAACACTAAAAATAGAATCTACTACATATATTC
TGTGCTTGTGGAATTATACTTTCTGTTTATGACTCAGCAGATTTTGGTATCTATGGTAATA
TATAGAGGATGTACGGAAAGAGTAGGAGAACTGATTTGTTATTACATTCAATATACCAAT
TCCAACATATGCAGTTTCCTGTCGAAGAGAAGCAAAATTCGGAAGATTTTTAACTATAT
AATGGACAATGAAGCAGAACATTTAAAACACGGAACAGCTGATGTTTATTTAAAATAC
GCCAAAGTGAACAGAAAAGTTATTGTACTTTTTCTAGTCCTTACAGGGGTAGCAGGCG
CTATTTGGTATATATTGGTCGTTAGAGATACCTTCTTCGCCGAAGAAAATGAAAATTGCC
TAATTTTGAGAGGCTTAAACTTTCAGATTTGGTATCCCTTCGATCTATTTAATCGATGTTA

CGTGATTACTTTACTAAATGACATTCTGATGTACACATCTGCAGTTGGTGTTCACATATA
CAATAAAATAAGTCCGGTGTCGTTTATGATTTACATTCTTGGGCAAATTAAGATACTACA
AGAGATGCTTAGGTGCATTGAGAAAGATGCTATAAGTATGCACGAATTGCAAGGAGAA
AAATATGAAGAAGCTATTTTGAAAAATATTAATAATTGTGTTAAGATGCATCAAGAAGTT
ATAAAGTTTATGGGTCTAATTGATAAAGGATGTAAAGAAATCGTTTTGATTGGATTTTTT
ACAAACTCGTTAGAGTTGGCAGCATTTGTTATTAAAGTTCTTATGGAAGAAGATGTATT
CGGGGCTTTAAGAACTATCGGTATATTATGCATGACAGTTACCCAATTATTTATGTTTTTC
TGGTTCGCAAACGAAATTAAAGTTGAGAGTACTTATATATCAGATGTTATTTACTACCAA
ACTAATTGGATAAGCTATGACAAACACGCTCGAAGGCATTTGTGGTTAATGATGATCAG
ATCTCAACGACCCCTTACTATTAGTGCTGCAGCTATTGGAGATATGTCTATTGATACTTTT
AAGAGGATCATAAAACTGTGCTACAGTATTGCAACGTTCTTTAAAACTGTATACATGTA
A

>McarOR17
ATGGTTAAACCTATAGAAACCGTTTGTTGCCGAACGACTTTAAAAATTCTTCGGACGTG
CTATATGTACCCAGAAGAGGGTAAGGAGCAAAATCCTGGCAAGTTGTTCCTATTGAAAT
GGTTGACGTTGATGATCTTAAGTTCAGTTACCTTCATTGGCAGTTTCCTACACCTTATAA
TAAGTTTGAAGGACGAAGACTATAGGCATTTGGATGTAGACTTTTCTATCACGTTATCC
ATGATAGCAACTTACATATTCACTTGCTTCTTCTTCGCCAGAGTTAAATTCGCCTCCAAA
TTTTACATGCACTTATCAAACTTAGAACGCTTGGAGAAACCTCTCGATTTCGAGAAGAA
GAACGAAAGATTAGAAAAGTTTGCCCTGTACCATTACATCTACATGGAACTACTAGTCG
CATCTCTGCTTCTGTTTTCCAATGTTATCAAAGGCGCCAAATGCAAACAAGAAAATCTG
GAATTCGACCTTCACGAGGTGTGTGGACTGTTTACGTATACGTGGATGCCTTTCGATATA
GATTACTTTCCTGTCAAGCAAATATACCTGTTCCTACAACTATTTGGCACCCATTATTTAT
ACTTGATAGCTGGCACGATGGCATGGACGGTGGTGGAGGCGATTCAACAAATTGTCTT
GAGGCTGCGCCACGCCAAGTACCTTTTCACGGAAGCGATAAAGGAGGTGGATCCCGT
GCTGCAGAGGCAAAAGTTCAATAGGGCCGTTCGATACCACGATGCCGTTTTAGGTTTG
GATGATCGCTTAAATGGAACATTTGGTGTATTTATGTTTACCCACCTGGGAATGACGGCT
CCTATATTGGGAACAGCATTTTTTGCCATATTACACGGTGGTTCTGGCTCCTCGCTATTC
ATTTGTTTGGGCTGGTTTATCGGTGTCTCAATGGATTGTTTTAGTGGACAGCATTTACAA
AATGAGAGCATCGATATCGCTAGAGCCCTGTATGACACCCAATGGTATAACTGTAGCCA
AGACATAAAAAGAGATGTTTTATTCGTGTTGATGAGATGCACAAAGCCGATGTACTTGA
AAGCTACTTCATTTGGGATTATGGACCGTGTGATGCTGCTTGGAGTTCTGAAAGCTACA
TACTCCTACATCGCTCTATTGACACAGACCCAATAA

>McarOR18
ATGGATGAAATTAAAGAAGAGGAGCCTTTCACGCACTCTTTAAAAATGCTGAATGTAAT
GGATGCTTTTCCATTAGAACATAATTTTTTCTCGAATGGGATCTTTTTTGTACGTTTTTGG
ATACTGAGGACAATGTCTTTCTGCATATCTTGCGTTTTACCTACCGTTCACATGGTAACA
TCAGTTAAAGATGGAATAAAACTTATCATAAGCGAAGACTTATCCGTTATTGTTGGTACC
ATGGTAAGCCTGATAACAACCTGCATATTCGTATTCAAAAGAAATAGTTGGTCGAAGTT
GCTCTCTGATATAGCAGACTTGAAAATATATGGAAGTTTCTCGGATTTTGATATTGTTAA
AGTGAAACTGAACCTATTTTCTAGAATATACTTTTGGTATTGCGTCAATGCGACGTTCGT
TTATGGCTCCGTATCGTTCATAGATACGTCTCAATGCGAAGAAATAAACAAATTAGGAA

AAGAGTGGCGTGAGGTTTGCGGTACATATTTACCAATGAGGTTGCCCTTTGACGCTAAT
ATACAATGGATGAGAGTATCAATTTTCTTCACACAGATGTTTTTCACTTTACTTTCATTA
GCACCTAGCGCACTTGCTTGCTCTATAATATTTCAGTCTACGGGATTTATTATAGCGCAC
ATTGAAAACCTGAAGAAACACCTGGTAGGAGCTTTTGATTCGACGGATGTGCAAGAGA
CATCTAACATATTGCGATACTGCATAAGTTATCACAATCACATCTTAAGGTTATCAGCAA
GACTACAAGATTTAGTAGGAACTAATATCAGTCACGTATTACTTATGTCGGCAGTAGTCT
TTGCCGGTATTGGAAACCAAATTTTAAAAACAAAACCTGTTGGAGGTACATTATATTTT
ATTGGGTATATGATAGCTTTATTCTTATTGTGCCACTCGGGGCAAAGGCTCATAGATGAG
ACAGCTTCTATTGGATCTGCAGCTTATAATTCAAAGTGGTACAAAGGCAATACGTCAAT
GATTCGAGACACTTTATTAATAATTTATCGAAGTCAAAAACCATGCACATTAGAAGTATT
GTCACTGGGAAGCTTAAATTATCCATTGTTTCTCTTGATAATTAAAACTTCATATAGCTAC
CTCACCCTACTTCAACAAACTGCTTAA

>McarOR19
ATGTTTAAAATTCAGAAGGGAGCACCCTTTTACTCCACCCTTCTAGCGTTAAGTTTGTT
TGGTCAAATTCCTGTGGAATTTAAAGAAACGTATTCAAAAAGTTTTCTAGTTAAATGCA
TTCTCTCGAGGTTTATTGGTTTTGTTCTCGTTTCAGTCGCACCAATATTGCAATACGTAA
TGGCCACTAAAGGAAGCATTGAAGTTGACATAAGCGAAAACATTTCTATAACAATCAG
TTCCATAGGAGCTCTATTGACAGGCACTTTCCTCACCTGCCAATACAAGAAGTGGCTCA
AATTTTTCGAGGATATCACGGATCACAAAGCGTTTGGGAAACCTCCCGACTACGAAGA
CCTTGTCAAGAATTTCAATCGCTTTTCGGCTTTCTACACTATTTATTGCACGGGATCAGT
CCCTGTCTACGCAGTAACAGTCTACTTCAATTCGATGAGATGTGATGAAGCAGCTCTTA
AGGCAGGATTTTTCTGTAAATCCTTTACACCAATATGGTTACCCATTGATGGGAGTAGCC
TTCAATTACGAGTTATCATATATATTGTTCAAATGCTTCTTGGAGGAACTATTGTATGTAC
AAGCGCGGTGATCAATTTCATGGTTTGGGAATCAACAGAAATGTTGATATCGCATATTA
ATAGTCTGAAGATTCATTTCAATAAAATCAGTGAGAAATCAACGGATAAGGAACGATCT
GAACAACTTGGCTTCTGTGTCCGGTACCATAATCATATACTAAGGTTGTCAAGTCGTTTA
AATGGGCTAATAAAATGGACATCTGGACATATGTCCCTCACAGCAGCATTAATTTTTGCC
TCGATTGGAAACCAAATATCTAATTCTAAGTCTGTGGGAGCATTTTTATACTTGATAGGG
TACGTTGGAGCATTATTTTTCATATGCCATGCTGGACAAAGGATTAAAGACGAGTTAAT
GTCTGTTGGTGACGCCGTTTACAATGCAGATTGGTATGCAACCGACGTCAAAACTATAA
GAAGCCTAAGATTTATAATAGCTAGGTGTCAAATACCATTCCATTATGAAGCCATTCCGT
TGGGCGTAGTGGATTATCCCCTATTTCTAATGATAATAAAGACCTCATATTCATACGTGAC
CCTATTAAGTCAAACTACATGA

>McarOR20
ATGATTAAGATAAGAGATATAGTTTGTTGCACAGTCAGTATAAAAATTCTTCAAGTATGT
TTTTTGTTCCCACTGAAAGGCAAAGAGCTGGAACCAAACTATTTGCGGGGATTTATTTT
CTTCTTTCTGATGGGTTTTTCTTCTCTCACTGTTATTGGGAGCTTTCTACACTTCATAATA
AGTATAAAGAATCACGTCTACTACCACATTGATTTAGATATGGCCATAATGATTTCAATG
TTTACCACCTATTCGTTCTCGATCGTCTTCTTCTTTAACATTAAAAGCGCCGTTCGTCTT
TACATGACTCTATCGGACTTCGATGAACACGGAAAACCCCGTAATTTCGACAAAAGAA
CAAAACTAATAGATAAAGTTGTCACTTACTATTATATCTATATCGAATTTTTAATAATATTT
ATGCTATCAACGTCTAATGTCAGCAGTAGCGGCAAATGTAAAAAAGAAAATGAAAAAT

ATGGCCTCAACGAAGTATGTGGACTGTTTTCGTACACGTGGATGCCTTTTGAAATAGAT
TATTATCCTGTCAAACAAATATATACGATCTGTCAGCTGGTGGGTACTCATTATCTAATTA
TCCTGGCTGGAGTGGTATCATGCTTGATGGCCGAGACGATGGAACAAATAATAACTAGA
ATACACCACGCCAGATATTTATTTCTTGAAGCGATAAAAGAGAAGGACTACGCAAAGC
AGAGACAAATGTTCAATACAGCTGTCCGCTATCATATTGGAGTTTTAGACTTGGAAGAC
CCACTGAATGAAACTTATGGCTTCTTCATGCTTACCCATCTCGCAATGACTGCTCCGATT
ATAGGAACTGCACTGTATTCAATTCTATATGGAGGATCTGGTTCATCTACGTTCATTTGT
CTAGGCTGGTTCATTGGTGTAATGAAGGATTGTTGTTGTGGACAACGTTTACAAAGTCA
GAGTAACACGGTTCCCATAGCTATATACGACAGTGAATGGTATACTTGTAATGAGGAGA
TAAAAAAAGATATTTTATTCGTACTGATGAGATGTAGACGACCCATGTATCCCAAAGCA
ATTTCTTTCGGAGTACTGGATCACGTCATGTTTCTTGGAGTTGTGAAAGCTGCCTATTCT
TACATTGCATTACTGAGTCAAACCACATAG

Table S7. Protein names and gene accession used in phylogenetic tree of IRs

Name	ID	Name	ID	Name	ID	Name	ID
AglaIR25a	XP_018574744.1	HparIR4	AVH87292.1	PstrIR3	ANQ46495.1	PakaIR6	QGW50648.1
AglaIR40a	XP_023310509.1	HparIR5	AVH87293.1	PstrIR4	ANQ46496.1	PakaIR7	QGW50649.1
AglaIR21a	XP_023313060.1	HparIR6	AVH87294.1	PstrIR5	ANQ46497.1	PakaIR8	QGW50650.1
AgerIR1	QNH68025.1	HparIR7	AVH87295.1	PstrIR6	ANQ46498.1	PakaIR9	QGW50651.1
AgerIR2	QNH68026.1	HparIR12	AVH87300.1	PstrIR7	ANQ46499.1	PakaIR10	QGW50652.1
AgerIR3	QNH68027.1	HparIR13	AVH87301.1	PaenIR7	APC94352.1	PakaIR11	QGW50653.1
CbowIR8a	ALR72538.1	HparIR14	AVH87302.1	PaenIR1	APC94347.1	PakaIR13	QGW50655.1
CbowIR6	ALR72535.1	HparIR15	AVH87303.1	PaenIR2	APC94349.1	PakaIR15	QGW50657.1
CbowIR5	ALR72540.1	HparIR19	AVH87307.1	PaenIR6	APC94350.1	PakaIR17	QGW50659.1
CbowIR75q	ALR72537.1	HparIR20	AVH87308.1	PaenIR3	APC94351.1	PakaIR20	QGW50662.1
CbowIR2	ALR72541.1	HparIR23	AVH87311.1	PaenIR8	APC94353.1	PakaIR21	QGW50663.1
TmolIR2	AJO62240.1	HparIR26	AVH87314.1	PakaIR1	QGW50643.1	PakaIR22	QGW50664.1
TmolIR5	AJO62243.1	HparIR1	AVH87289.1	PakaIR2	QGW50644.1	PakaIR18	QGW50660.1
TmolIR6	AJO62244.1	HparIR17	AVH87305.1	PakaIR3	QGW50645.1	PakaIR16	QGW50658.1
HparIR27	AVH87315.1	PstrIR1	ANQ46493.1	PakaIR4	QGW50646.1		
HparIR3	AVH87291.1	PstrIR2	ANQ46494.1	PakaIR5	QGW50647.1		

Table S8. Protein names and gene accession used in phylogenetic tree of SNMPs

Name	ID	Name	ID	Name	ID	Name	ID
TmolSNMP1	AJO62245.1	LdecSNMP1	XP_023018522.1	HparSNMP1	AVM18969.1	ItypSNMP2	JAA74403.1
TmolSNMP2	AJO62246.1	TcasSNMP1	EFA02899.2	HparSNMP2	AVM18970.1	MaltSNMP1	AIX97076.1
PstrSNMP2	ANQ46505.1	TcasSNMP2	KYB28339.1	AgamSNMP1	P86905.1	AglaSNMP2	XP_018566911.1
PstrSNMP1	ANQ46504.1	BmorSNMP1	NP_001037186.1	AgamSNMP2	Q7Q6R1.5		
LdecSNMP2	XP_023025981.1	BmorSNMP2	XP_004933211.2	ItypSNMP1	JAA74404.1		
